# Prognostic impact of solid tumor component diameter in early-stage non-small cell lung carcinoma treated with intensity-modulated fractionated radiotherapy: a retrospective analysis impact of solid tumor component diameter in NSCLC treated with IMRT

**DOI:** 10.1259/bjr.20191027

**Published:** 2020-04-13

**Authors:** Tomohiro Itonaga, Ryuji Mikami, Mitsuru Okubo, Tatsuhiko Saito, Sachika Shiraishi, Shinji Sugahara, Koichi Tokuuye, Kazuhiro Saito

**Affiliations:** 1 Department of Radiology, Tokyo Medical University Hospital, 6-7-1 Nishi-shinjyuku, Shinjyuku, Tokyo, Japan

## Abstract

**Objective:**

To investigate the suitability of the new diameter-based subgroupings of the eighth edition Tumor Node Metastasis (TNM) classification system regarding radiotherapy treatment for early-stage non-small-cell lung cancer (NSCLC), we retrospectively re-analyzed the clinical data of patients treated with intensity-modulated radiotherapy using non-coplanar beams (ncIMRT) for Stage I NSCLC.

**Methods:**

Between March 2011 and March 2018, 92 patients with 94 tumors who were diagnosed with Stage I NSCLC according to the seventh edition TNM classification system were enrolled and underwent ncIMRT of 75 Gy in 30 fractions. Local control (LC), progression-free survival (PFS), and overall survival (OS) were retrospectively investigated according to the T-classification subdivisions of the eighth edition and maximal solid tumor component diameter.

**Results:**

The median follow-up period was 32.5 months. The median maximum tumor and solid tumor component diameters were 22 mm and 18 mm, respectively. 3-year LC, PFS, and OS rates were 84.1%, 69.4%, and 85.3%, respectively. The 3-year LC rates were 91.0 and 76.8% in the groups with tumor diameter ≤2 cm and >2 cm, corresponding to the T1c and T1b subdivisions of the eighth edition, respectively (*p* = 0.24). In the ≤2 cm and >2 cm solid tumor component groups, the 3 year LC rates were 93.6 and 63.2%, respectively, which were significantly different (*p* = 0.007).

**Conclusion:**

LC rates after radiotherapy in patients with Stage I NSCLC were correlated with solid tumor component diameter. High LC rates in patients with solid tumor components <2 cm in diameter were associated with high PFS and OS rates.

**Advances in knowledge:**

This study suggests that the eighth edition TNM classification system, which focuses on solid tumor components rather than tumor diameter, can be applied to radiotherapy.

## Background

Because metastases to mediastinal and hilar lymph nodes are uncommon in early-stage non-small cell lung cancer (NSCLC), high dose concentration in the primary lesion may increase chances for a complete cure.^1^ For inoperable Stage I NSCLC, the sophisticated method of stereotactic body radiotherapy has shown higher local control (LC) and lower toxicity rates compared to those of conventional radiation therapies.^2–6^ However, large tumors still recurred, even after high dose radiation therapy.

The eighth edition of the Tumor Node Metastasis (TNM) staging system for NSCLC was released by the International Association for the Study of Lung Cancer (IASLC)^7^ based on 70,967 patients with NSCLC between 1999 and 2010. Recent advances in high-resolution CT allow whole tumor and solid tumor component diameters to be objectively measured in patients with early-stage lung cancer. Therefore, in the eighth edition system, the T-classification was subdivided by 1 cm increments of the maximal tumor diameter.

To investigate the efficacy of using the eighth edition TNM classification system and solid tumor component diameter for planning radiotherapy treatment, we retrospectively analyzed clinical outcomes according to the whole tumor and solid tumor component diameters. As many patients had chronic pulmonary diseases in our studies, we reduced the fraction dose to minimize treatment-related toxicities: Non-coplanar intensity-modulated radiotherapy (ncIMRT) with 75 Gy in daily doses of 2.5 Gy, which corresponds to 93.8 Gy in biologically effective dose calculation when α/β value was assumed to be 10, was used.

## Patients and methods

The ethical committee of Tokyo Medical University Hospital in Tokyo, Japan approved the original Phase II study (IRB number 1625) and this retrospective analysis (IRB number SH4121). All patients agreed to use the clinical data for research at the start of radiotherapy. The eligibility criteria were as previously reported^8^ ; briefly: (1) age of 20 years or older, (2) performance status of 0 or 1 according to the World Health Organization guidelines, (3) diagnosed with NSCLC by cytology or histology, or clinically diagnosed with NSCLC by findings on positron emission tomography (PET-CT) or a tumor that had increased by more than 25% within 2 months on CT when a histological diagnosis was not made, (4) clinical stage of T1-2aN0M0 according to the seventh UIBC TNM classification by CT or PET-CT taken within the past 40 days, (5) medically inoperable conditions determined by the cancer board, which consisted of thoracic surgeons, medical oncologists, radiation oncologists, and diagnostic radiologists.

### Intensity-modulated radiotherapy

The details of the treatment procedure are as previously described.^8^ In short, all patients were immobilized in the supine position using a body-fixed shell system (Pelvicast; Orfit Industries n.v., Wijnegem, Belgium) to reduce tumor motion caused by pressing the abdomen. The clinical target volume was defined as the gross tumor volume plus a 0.5 cm margin in all directions, and the internal target volume was defined as the clinical target volume image of the exhalation phase overlapped with that of the inhalation phase. The planning target volume was defined as the internal target volume plus a uniform margin of 0.5 cm in all directions. The prescribed dose was determined to be 75 Gy in 30 fractions covering 95% of the planning target volume (D95). The IMRT plan was created with a treatment planning system (Xio v. 4.6 system, Elekta AB, Stockholm, Sweden). The treatment plan was verified for clinical use with a three-dimensional radiation detector (Delta 4, ScandiDos AB, Uppsala, Sweden). Chemotherapy was not performed during radiotherapy. CT to verify tumor location and adjust the patient’s position was taken weekly or, if needed, daily.

### Computed tomography

Images of the lung tumors were taken using a 64 multislice CT (Light Speed VCT, GE Medical Systems, Milwaukee, WI,) and 16 multislice CT (Aquilion LB, Toshiba Medical Systems, Tokyo, Japan). Raw CT data were reconstructed into an axial CT image with 2.5 mm slice thicknesses according to the JCOG0201 definition.^9^ The CT image was displayed with a window level of −600 Hounsfield unit (HU) and a window width of 2000 HU as the lung image. Based on the JCOG0201 study, ground-glass opacity was defined as an area of a slight, homogenous increase in density that did not obscure any underlying vascular markings, and was considered to be tumor.^9^ The solid tumor component was defined as an area of increased opacification that completely obscured any underlying vascular markings. The diameters of the whole tumor and solid tumor component were measured by thin-slice CT using the SYNAPSE VINCENT software program (Fujifilm Medical, Tokyo, Japan). Peripheral and central types were distinguished to determine whether the tumor was located in the “no-fly zone”—2 cm around the proximal airway, as defined in the Radiation Therapy Oncology Group 0236 trial.^10^ Measurements of tumor diameter and tumor location were determined from the CT data without accompanying clinical data by two different radiologists.

### Follow-up

Patients were followed-up every 3 months after the completion of radiation treatment. They received the usual medical consultation and underwent chest CT scans every 3 months for 2 years and then every 6 months thereafter. Recurrence has been determined when recurrent tumor was demonstrated pathologically, or by PET-CT findings, or if a tumor increased by more than 25% in length in follow-up CT. The final judgment regarding recurrence was made by the cancer board held every week.

### Statistical analysis

The LC, progression-free survival (PFS), and overall survival (OS) rates were calculated from the start date of ncIMRT using the Kaplan–Meier method. To further analyze the clinical data, we created a cut-off value of 2 cm for the maximal tumor diameter according to the eighth edition TNM classification system. The log-rank test was used to compare outcomes between the subsets of patients analyzed. Multivariate analysis with the Cox proportional hazards model was used to determine prognostic factors among the potential factors identified by univariate analyses. All statistical analyses were performed as two-tailed, and a *p*-value of <0.05 represented statistical significance. R software (v. 3.1.0, R Foundation for Statistical Computing, Vienna, Austria) was used for all statistical calculations.

## Results

### Patient and tumor characteristics

Between March 2011 and March 2018, a total of 92 patients with 94 tumors were diagnosed with Stage I NSCLC (T1–2aN0M0, seventh edition UICC TNM classification) and enrolled in this study. Data were collected until the last day of March 2019. The median follow-up period was 32.5 months (range, 3.8–95.8). Patient and tumor characteristics are summarized in Table 1. Of the 92 patients, 62 were male and 30 were female, and the median age was 79 years (range, 49–93). Of the 94 tumors, there were 26 adenocarcinomas, 16 squamous cell carcinomas, 6 NSCLC, and the remaining 48 tumors were diagnosed by PET-CT and/or 25% enlarged over a 2 month period on CT. The median maximal whole tumor and solid tumor component diameters were 22 mm (range, 10–50) and 18 mm (range, 0–48), respectively. 55 tumors were larger than 2 cm in maximal diameter and 39 smaller than 2 cm; 37 tumors had a maximal solid tumor component larger than 2 cm and 57 smaller than 2 cm. The results of re-staging after the transition from the seventh to the eighth edition TNM classification system are shown in Table 2. At the time of analysis, 16 patients (17.4%) died, and 16 tumors (17%) recurred locally. Of the 16 deceased, 8 died from lung cancer progression, 2 died from other malignant diseases, 2 died from community-acquired pneumonia, and the remaining 4 patients died of cerebrovascular diseases.

**Table 1.** Patient and tumor characteristics

Factors		No
Sex	Male	62
	Female	30
Age	Median	79
	Range	49–93
Location	Peripheral	76
	Central	18
Pathology	Adenocarcinoma	26
	Squamous cell carcinoma	14
	NSCLC	6
	Pathology unproven	48
Maximum tumor diameter	Median	22
	Range	10–50
Solid tumor component diameter	Median	18
	Range	0–43

NSCLC, non-small cell lung carcinoma;

**Table 2.** Re-staging after the transition from the seventh to the eighth edition of the TNM classification system

	TNM seventh	TNM eighth
Tis	-	3
T1a	38	14
T1b	32	40
T1c	-	20
T2a	24	15
T2b	-	2

TNM, Tumor Node Metastasis.

### Survival outcomes

**Figure 1.**
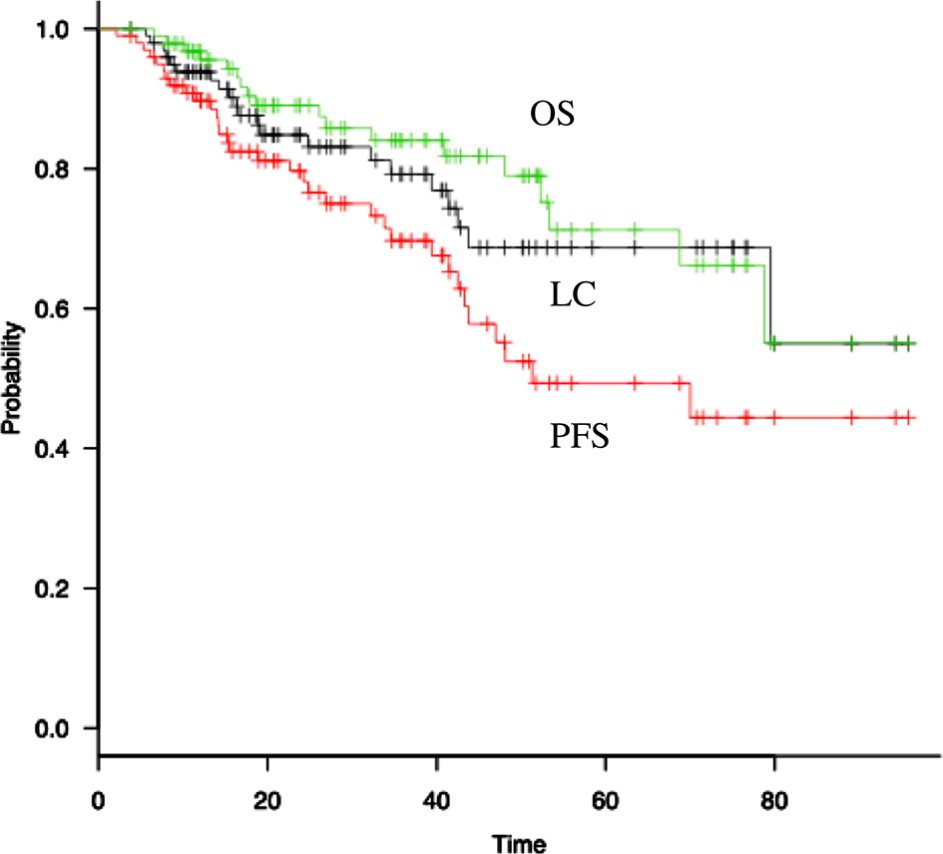
LC, PFS, and OS after the completion of IMRT, estimated using the Kaplan–Meier method, are shown. IMRT, intensity-modulated radiotherapy; LC, local control; OS, overall survival; PFS, progression-free survival.

The 3 year LC, PFS, and OS rates were 84.1% [95% confidence interval (CI), 72.5–91.1], 69.4% (95% CI, 57.1–78.9), and 85.2% (95% CI, 74.7–91.6), respectively (Figure 1). The 3 year LC rates for tumors either ≤2 cm or >2 cm in maximal tumor diameter were 91.0% (95% CI, 74.6–97.0) and 76.8% (95% CI, 57.1–88.3), respectively, but were not significantly different (*p* = 0.24). In contrast, the 3 year LC rates for solid tumor component diameters either ≤2 cm or >2 cm were 93.6% (95% CI, 0.813–0.979) and 63.2% (95% CI, 0.363–0.813), respectively, which was a significant difference (*p* = 0.007). Concerning PFS, the 3 year PFS rates were 81.3% (95% CI, 62.5–91.2%) *vs* 59.7% (95% CI, 41.9–73.6%) for tumors either ≤2 cm or >2 cm in maximal diameter, respectively, which was statistically insignificant (*p* = 0.16), and 82.1% (95% CI, 66.9–90.7) *vs* 47.2% (95% CI, 25.7–66.0) for tumors either ≤2 cm or >2 cm in maximal solid tumor component diameter, respectively, which was significant (*p* = 0.002). The 3 year OS rates were 88.7% (95% CI, 72.6–95.6) *vs* 81.4% (95% CI, 64.0–90.9) for tumors either ≤2 cm or >2 cm in maximal diameter, respectively, and 87.6% (95% CI, 74.3–94.3) *vs* 80.3% (95% CI, 57.2–91.7) for tumors either ≤2 cm or >2 cm in solid tumor component diameter, respectively, which were statistically insignificant (*p* = 0.376 and 0.267, respectively).

### Univariate and multivariate analyses

Tables 3 and 4 show the univariate and multivariate analyses, respectively, of putative prognostic factors for LC, PFS, and OS. After univariate analysis, pathology and solid tumor component diameter were significantly associated with LC. After multivariate analysis, we determined tumor location, age, and tumor size, which were significant clinical factors in previous studies, to be independent prognostic factors. Regarding LC, solid tumor component diameter >2 cm (hazard ratio, 3.92 ; 95% CI, 1.33–11.54; *p* = 0.01) was the only significant risk factor among these factors by multivariate analysis.

**Table 3.** Univariate analysis using the Cox proportional hazard model

	LC		PFS		OS	
**Factor**	HR (95% CI)	*p*-value	HR (95% CI)	*p*-value	HR (95% CI)	*p*-value
**Age**	1.02 (0.95–1.08)	0.63	1.00 (0.96–1.05)	0.87	1.08 (1.00–1.16)	^*a*^0.04
**Sex** **Male *vs* Female**	3.26 (0.92–11.64)	0.07	4.58 (1.72–12.15)	^*a*^0.002	5.42 (1.20–24.42)	^*a*^0.03
**Pathology** **Proven *vs* Unproven**	3.28 (1.12–9.61)	^*a*^0.03	1.27 (0.63–2.56)	0.50	2.24 (0.80–6.27)	0.13
**Location** **Central *vs* Peripheral**	0.90 (0.25–3.15)	0.86	0.78 (0.32–1.90)	0.58	1.42 (0.49–4.14)	0.52
**Maximum tumor diameter** **>20** mm	1.84 (0.66–5.15)	0.25	1.65 (0.81–3.36	0.16	1.59 (0.57–4.44)	0.38
**Solid tumor component diameter >20** mm	3.92 (1.35–11.37)	^*a*^0.01	3.10 (1.49–6.44)	^*a*^0.002	1.85 (0.61–5.57)	0.27

CI, confidence interval;HR, hazard ratio;LC, local control; OS, overall survival; PFS, progression-free survival.

a *p* < 0

**Table 4.** Multivariate analysis using the Cox proportional hazard model

	LC		PFS		OS	
**Factor**	HR (95% CI)	*p*-value	HR (95% CI)	*p*-value	HR (95% CI)	*p*-value
**Age**	1.00 (0.94–1.06)	0.97	0.99 (0.95–1.03)	1.03	1.07 (1.00–1.15)	0.06
**Tumor location** **Central *vs* Peripheral**	0.85 (0.24–2.98)	0.79	0.76 (0.31–1.84)	0.54	1.32 (0.43–3.82)	0.66
**Solid tumor component diameter >20** mm	3.92 (1.33–11.54)	*0.01	3.20 (1.52–6.74)	*0.002	1.43 (0.46–4.39)	0.54

CI, confidence interval;HR, hazard ratio;LC, local control; OS, overall survival; PFS, progression-free survival.

## Discussion

Prognosis prediction is essential for determining the proper treatment modality for lung cancer. TNM classification has played the most important role in predicting prognosis so far, and treatment methods have been decided accordingly. The classification system has recently been revised according to an increase in medical knowledge and advances in medical treatments. In lung cancer, the T-characteristic of the TNM classification system has been updated considerably between the seventh and eighth editions based on the IASLC database. Notably, in the eighth edition, tumor size has been subdivided by 1 cm incremental increases.

In the IASLC database, approximately 85% of enrolled patients have undergone surgical treatment, and it is unclear whether this includes patients who have received radiotherapy. Although a pathological diagnosis is essential to determine the proper strategy for radiotherapy treatment, the procedures necessary for pathological confirmation can be challenging due to the patients’ condition or refusal. Advances in medical imaging technologies, such as PET and high-resolution CT, make it possible to diagnose lung cancer with high accuracy. Because of this, we analyzed the data of patients treated with ncIMRT for Stage I NSCLC to clarify the usefulness of the eighth edition of TNM classification.

As CT and its related technologies progress significantly, we can measure the tumor diameter and solid tumor component diameter more objectively using imaging software. Recently, the solid tumor component has been recognized by several studies as an important prognostic factor in lung cancer.^9,11–13^ In this study, we used treatment planning CT with determined conditions and measured the maximal tumor diameter and maximal solid tumor component using Synapse Vincent software to obtain accurate data.

Eriguchi et al retrospectively reported that the 3 year LC and PFS rates after stereotactic body radiotherapy in patients with lung tumors that had a maximal solid tumor component to tumor diameter ratio less than 0.5 were 100%.^14^ In our study, the maximal solid tumor component diameter predicted LC and PFS better than the maximal tumor diameter. There are few studies showing the association between tumor radiological features and prognosis after radiotherapy in early-stage NSCLC. Further studies are needed to explore these associations.

Herein, we reported the outcomes of ncIMRT for Stage I NSCLC, which were comparable with the results of other studies.^10,15–17^ In this study, we analyzed our data according to the newly subdivided T-classification of the eighth edition TNM classification system. When cut-off lines were set to either 2 or 3 cm for maximal tumor diameter, there were no significant differences between the two groups. In contrast, when divided by solid tumor component diameter, significant differences were seen between groups. This result shows that the solid tumor component diameter has a greater effect on prognosis than the maximal tumor diameter after radiotherapy treatment.

Concerning other prognostic factors, tumor location, tumor size, sex, and age are known to be prognostic factors.^18–20^ In this study, the clinical data of the primary tumors was the same as that of any peripheral tumors. For T1 tumors located near high-risk structures such as the esophagus and bronchial tree, it may not be necessary to select a different treatment schedule in our setting.

From these data, a classification system based on the tumor solid tumor component may be an alternative to one based on the maximal tumor diameter. Due to insufficient information regarding pathological conditions in our protocol, the results of this study should be interpreted carefully. Treatment planning, including administering a higher dose to the solid tumor component, may be effective for the treatment of NSCLC.

The limitations of this study are its retrospective nature, which has an inherent potential bias. Insufficient pathological data are also a limitation of this study.

## Conclusion

LC rates after radiotherapy in patients with early-stage NSCLC were associated with maximum solid tumor component diameter rather than maximum tumor diameter.
